# Regulation of the *cd38 *promoter in human airway smooth muscle cells by TNF-α and dexamethasone

**DOI:** 10.1186/1465-9921-9-26

**Published:** 2008-03-14

**Authors:** Krishnaswamy G Tirumurugaan, Bit Na Kang, Reynold A Panettieri, Douglas N Foster, Timothy F Walseth, Mathur S Kannan

**Affiliations:** 1 Department of Veterinary and Biomedical Sciences, College of Veterinary Medicine, University of Minnesota, St. Paul, MN, USA; 2 Department of Medicine, University of Pennsylvania, Philadelphia, PA, USA; 3 Department of Animal Science, University of Minnesota, St. Paul, MN, USA; 4 Department of Pharmacology, College of Medicine, University of Minnesota, Minneapolis, MN, USA; 5 Department of Pediatrics, College of Medicine, University of Minnesota, Minneapolis, MN, USA

## Abstract

**Background:**

CD38 is expressed in human airway smooth muscle (HASM) cells, regulates intracellular calcium, and its expression is augmented by tumor necrosis factor alpha (TNF-α). CD38 has a role in airway hyperresponsiveness, a hallmark of asthma, since deficient mice develop attenuated airway hyperresponsiveness compared to wild-type mice following intranasal challenges with cytokines such as IL-13 and TNF-α. Regulation of CD38 expression in HASM cells involves the transcription factor NF-κB, and glucocorticoids inhibit this expression through NF-κB-dependent and -independent mechanisms. In this study, we determined whether the transcriptional regulation of CD38 expression in HASM cells involves response elements within the promoter region of this gene.

**Methods:**

We cloned a putative 3 kb promoter fragment of the human *cd38 *gene into pGL3 basic vector in front of a luciferase reporter gene. Sequence analysis of the putative *cd38 *promoter region revealed one NF-κB and several AP-1 and glucocorticoid response element (GRE) motifs. HASM cells were transfected with the 3 kb promoter, a 1.8 kb truncated promoter that lacks the NF-κB and some of the AP-1 sites, or the promoter with mutations of the NF-κB and/or AP-1 sites. Using the electrophoretic mobility shift assays, we determined the binding of nuclear proteins to oligonucleotides encoding the putative *cd38 *NF-κB, AP-1, and GRE sites, and the specificity of this binding was confirmed by gel supershift analysis with appropriate antibodies.

**Results:**

TNF-α induced a two-fold activation of the 3 kb promoter following its transfection into HASM cells. In cells transfected with the 1.8 kb promoter or promoter constructs lacking NF-κB and/or AP-1 sites or in the presence of dexamethasone, there was no induction in the presence of TNF-α. The binding of nuclear proteins to oligonucleotides encoding the putative *cd38 *NF-κB site and some of the six AP-1 sites was increased by TNF-α, and to some of the putative *cd38 *GREs by dexamethasone.

**Conclusion:**

The EMSA results and the cd38 promoter-reporter assays confirm the functional role of NF-κB, AP-1 and GREs in the cd38 promoter in the transcriptional regulation of CD38.

## Background

CD38 is a pleiotropic protein that has enzymatic and receptor functions [1-3]. It is a ~45-kDa glycosylated transmembrane protein, with an extracellular domain that has an enzyme activity which generates cyclic ADP-ribose (cADPR) and ADPR from nicotinamide adenine dinucleotide (NAD) [1]. CD38 is expressed in different cells including airway smooth muscle (ASM) cells, where its expression is confined to the plasma membrane [4]. In ASM cells, CD38/cADPR signaling has a role in the regulation of intracellular calcium ([Ca^2+^]_i_) [5-7]. Previous studies from our laboratory showed that CD38 expression and its enzymatic activities are augmented by TNF-α and IL-13, cytokines that are implicated in the pathogenesis of inflammatory airway diseases such as asthma [5,8]. The regulation of CD38 expression by TNF-α requires NF-κB activation and involves MAPK signaling in ASM cells [9,10].

Glucocorticoids are used in the treatment of asthma [11] which regulate gene expression via the glucocorticoid receptor (GR)[12]. Upon activation by ligand binding, the GR translocates to the nucleus and acts either as a transcription factor or as an inhibitor of transcription factors such as NF-κB or AP-1. We have previously shown that TNF-α-induced CD38 expression in ASM cells is inhibited by glucocorticoids through a mechanism that involves decreased NF-κB activation [9].

Regulation of the CD38 gene has also been investigated in human myeloid cells [13]. In these cells, CD38 expression is induced by retinoic acid through the retinoic acid response element located within the first intron of the *cd38 *gene. Response elements for other transcription factors, including AP-1 have been described in other cell systems, including osteoblasts and osteoclasts [14] and in these cell lines, TNF-α-induced activation of a *cd38 *promoter fragment requires an intact AP-1 site. Sequence analysis of a 3 kb putative *cd38 *promoter fragment (GenBank Accession # DQ091293) cloned from a human erythropoietic cell line (K562 cells) in our laboratory revealed binding sites for NF-κB, AP-1, and GR (summarized in Table 1). To determine whether CD38 expression in human ASM cells is regulated by TNF-α and glucocorticoid response elements (GREs), we measured the binding of transcription factors and the GR to their respective putative sites within this promoter region. Our results demonstrate that TNF-α causes increased binding to the NF-κB site and to some of the AP-1 sites, and that mutations in either of the binding sites abolish promoter activation. Dexamethasone increases the binding of GR to some of the GRE sites within the promoter and abolishes promoter activation induced by TNF-α. These results demonstrate that TNF-α regulates CD38 expression transcriptionally through NF-κB and AP-1, and glucocorticoids decrease this expression possibly by binding to GREs within the promoter and/or also by decreased NF-κB- and AP-1-mediated transcription.

**Table 1:** Putative binding sites for AP-1, NF-B and GRE in the *cd38 *promoter.

***NF-B binding site***	***Location***	***Designator***	***References***
GGGATTCCTC	-1728 to -1719	NF-CD38	(46)
***AP-1 sites***	***Location***	***Designator***	***References***
TGAATCA	-2915 to -2909	AP-1–6	(47,48)
TTGGTCA	-2835 to -2829	AP-1–5	(49,50)
TTGACTCAT	-2798 to -2789	AP-1–4	(51)
AACTACA	-1041 to -1035	AP-1–3	(52)
TGCCTCA	-993 to -987	AP-1–2	(49)
TGAGGCA	-151 to -145	AP-1–1	(49)
***GRE sites***	***Location***	***Designator***	***References***
TGTTCT	-2662 to -2658	GRE-4	(53)
TGTTCT	-1398 to -1393	GRE-3	(53)
TGTTCT	-1069 to -1063	GRE-2	(53)
TGTTCT	-881 to -875	GRE-1	(53)

## Methods

### Materials

Tris base, glucose, HEPES and TNF-α were purchased from Sigma Chemical (St. Louis, MO). Hanks' balanced salt solution (HBSS) and Dulbecco's modified Eagle's medium (DMEM), Trizol, Lipofectamine™ 2000, Superscript III reverse transcriptase and the 1 kb DNA ladder were obtained from Invitrogen (Carlsbad, CA). Dual-Luciferase Reporter assay system, pGL3 basic vector, pRL-TK plasmid, GoTaq^R ^Green Master Mix and EMSA kit were purchased from Promega (Madison, WI). QuickChange Site-Directed Mutagenesis kit was obtained from Stratagene (La Jolla, CA). The nuclear extraction kit was purchased from Active Motif (Carlsbad, CA). Recombinant human glucocorticoid receptor protein (RP-500) was obtained from Affinity Bioreagents (Golden, CO). Antibodies for p65 or p50 subunit of NF-κB, *c-jun *and *c-fos *were purchased from Santa Cruz Biotechnology (Santa Cruz, CA).

### Promoter-luciferase reporter constructs and site directed mutagenesis

Genomic DNA was isolated from the human erythroleukemia cell line K562 and approximately 3 kb of the *cd38 *promoter was amplified by PCR using the following primers: 3181F 5'-TGATGCCTCCTGTTGGGGGTCTA-3' and 3181R 5'-CGGGAAAGCGCTTGGTGGTG-3' (GenBank Acc. No. DQ091293). The reverse primer (3181R) was phosphorylated using T4 polynucleotide kinase and PCR was performed under the following conditions: 94°C for 3 min denaturing, then 30 cycles of 94°C for 50 s, 59.6°C for 50 s, 72°C for 90 s, and a final extension at 72°C for 10 min to yield a 3240 bp fragment. A truncated 1.8 kb promoter was also amplified employing the same PCR program with annealing at 60°C using the primer pairs 1378F 5'-GCATGCATATGTTCATTGTAGCACTAT-3' and 3181R 5'-CGGGAAAGCGCTTGGTGGTG-3' which was phosphorylated using T4 polynucleotide kinase. The resulting 3 kb and 1.8 kb PCR fragments were gel purified, cloned into pCR 3.1 Uni vector (Invitrogen) and the reverse orientation was confirmed by sequencing at the Advanced Genetic Analysis Center, University of Minnesota. The 3 kb and 1.8 kb (truncated) positive clones were digested with *Hin*dIII/*Eco*RV and ligated into *Sma*I/*Hin*dIII digested pGL3 basic vector (Promega, WI, USA). This enabled cloning of the larger and truncated promoter fragments in the forward orientation to drive the expression of the luciferase reporter gene. The 3 kb and the truncated *cd38 *promoter fragments in the pGL3 basic vector were confirmed by nucleotide sequence analysis. To mutate the putative NF-κB and AP-1 binding sites, primers for mutated NF-κB and AP-1 binding sites were designed (Table 2). Putative binding sites are underlined and mutated sequences are shown in bold font. Mutations of the putative NF-κB or AP-1 binding sites in the promoter constructs were performed by the QuickChange Site-Directed Mutagenesis Method (Strategene, La Jolla, CA) using Pfu Turbo polymerase. Template DNAs were digested with the methylation-dependent restriction enzyme *Dpn*I. Bacteria were then transformed with *Dpn*I-digested DNA, and the cloned mutated constructs were confirmed by sequencing.

**Table 2:** Sequences of the primers for the *cd38 *putative NF-κB and AP1–4 binding sites.

**NFκB-mut-F**	5'-GTGGAAGACAGTATGG**C**GATTCCTCAAAGATCTAGAACC-3'	39 bp
**NFκB-mut-R**	5'-GGTTCTAGATCTTTGAGGAATC**G**CCATACTGTCTTCCAC-3'	39 bp
**AP1–4-mut-F**	5'-CTTGGCATCATCTTTGACT**TG**TCTCTTTCTTGCAAATGC-3'	39 bp
**AP1–4-mut-R**	5'-GCATTTGCAAGAAAGAGA**CA**AGTCAAAGATGATGCCAAG-3'	39 bp

The putative NF-κB and AP1–4 binding sites are underlined and the mutated sequences are shown in bold font.

### Sequence analysis of the cd38 promoter

The GeneQuest module of Lasergene 6.0 program from DNASTAR was used to identify the potential transcription factor binding sites in the *cd38 *promoter. The 3 kb sequence of the *cd38 *promoter was analyzed using GeneQuest for the potential transcription factor binding sites using tfd.dat file. Analysis revealed six AP-1 binding sites, one NF-κB binding site and four GRE binding sites within the *cd38 *promoter. The putative transcription factor binding sites on the *cd38 *promoter are shown in Table 1.

### Human Airway Smooth Muscle Cell culture

Human airway smooth muscle (HASM) isolated from the trachealis muscle and propagated as described previously [9,10]. were used in this study. The cells were plated at a density of 1.0 × 10^4 ^cells/cm^2 ^and were cultured in DMEM supplemented with 10% FBS, 2 mM L-glutamine, 100 U/ml of penicillin, 0.1 mg/ml of streptomycin, and 0.25 μg/ml of amphotericin B. HASM cells were transfected as described below, then 24 hrs following transfection they were growth-arrested by maintaining them for at least 24 hrs in arresting medium containing no serum, but in the presence of transferrin and insulin prior to TNF-α (50 ng/ml) or dexamethasone (1 μM) treatment and measurement of luciferase reporter activity.

### DNA Transfections

Transient transfections were performed with Lipofectamine™ 2000 according to the manufacturer's instructions. Cells (0.5–1 × 10^5^) in 500 μl of growth medium without antibiotics were plated one day before transfection. For the transfection, 0.8 μg of the vector DNA and 2 μl of Lipofectamine™ 2000 in 50 μl of Opti-MEM^® ^were mixed gently and incubated for 5 min at room temperature. Diluted DNA and lipofectamine were mixed and incubated for 20 min at room temperature to form complexes which were added to each well, and incubated at 37°C for 6 hrs. The cells were growth-arrested 24 hrs following transfection before exposing to TNF-α and dexamethasone. The cells were collected for luciferase reporter activity (described below).

### Luciferase reporter gene transactivation assay

Reporter gene assays were performed 24 hrs after transfection. Cell lysates were subjected to the Dual-Luciferase Reporter assay system and luciferase activities were measured with a luminometer (Lumat LB9507; Berthold). Cells were washed twice with phosphate-buffered saline (PBS) with no calcium and magnesium, and covered (0.1 ml/well) with Passive Lysis Buffer (Promega). The cells were then scraped, the lysate transferred to microcentrifuge tubes, which was mixed by vortexing for 15 s, then passed a few times through a needle and used for the reporter assay. A 20 μl aliquot of the lysate was mixed with 100 μl of luciferase assay reagent and placed in a luminometer to measure the firefly luciferase activity. The fluorescence was quenched by the addition of the Stop and Glo buffer and Renilla luciferase activity was measured after a 2 second delay. Firefly luciferase activities were normalized to *Renilla *luciferase activity to account for transfection efficiency. Samples were analyzed in triplicate and the experiment was repeated at least twice.

### Nuclear protein extraction

Nuclear extracts were prepared from growth-arrested HASM cells at confluence. The media were aspirated and washed with ice-cold PBS containing phosphatase inhibitors and the cells were scraped in 3 ml of the same buffer. The cells were pelletted by centrifugation at 1000 × *g *for 5 minutes and the supernatant discarded. The cells were resuspended in 500 μl 1× hypotonic buffer by pipetting several times, transferred to a chilled microcentrifuge tube and incubated for 15 mins on ice. Detergent (25 μl) was added, vortexed for 10 sec and pelleted by centrifugation at 14,000 × *g *for 30 sec at 4°C. The supernatant was removed and the nuclear pellet was resuspended in 50 μl of complete lysis buffer and vortexed for 10 sec. The mixture was incubated on ice for 30 min, vortexed briefly and pelleted at 14,000 × *g *for 10 min at 4°C. The supernatant (nuclear fraction) was aliquoted, protein content measured and stored at -80°C until use.

### Electrophoretic mobility shift assay (EMSA)

The protein concentration of the nuclear extract was quantitated using the Bradford protein assay (Bio-Rad, Hercules, CA). EMSA was performed as described earlier [9,10]. The double-stranded oligonucleotides containing the consensus binding sites for NF-κB, AP-1, GRE and the putative *cd38 *binding sites (as shown in Table 3) were labeled with [γ-^32^P]ATP (3,000 Ci/mmol at 10 mCi/ml) by T4 Polynucleotide Kinase (Promega, Madison, WI). Nuclear extracts (5 μg) from HASM cells or 1 μg of recombinant human GR protein were incubated for 30 min at room temperature with 0.4 pmol of double-stranded^32^P-labeled oligonucleotide containing the consensus binding sites in a total volume of 10 μl in a buffer containing 20% glycerol, 5 mM MgCl_2_, 2.5 mM EDTA, 2.5 mM DTT, 250 mM NaCl, 50 mM Tris-HCl (pH 7.5), and 0.25 mg/ml poly (dI-dC). After 30 min at room temperature, samples were separated on a nonreducing 4% polyacyrlamide gel using 0.5 M TBE buffer. The gels were dried and autoradiography carried out with intensifying screens at -70°C. To confirm specificity of the EMSA, competition assays were performed with a 100-fold excess of unlabeled NF-κB or AP-1 probe, or the SP-1 probe as a nonspecific competitor. Gel super shift assays were performed to confirm the specificity of the EMSA using anti-p65 or -p50 subunit of NF-κB, and anti-*c-jun *and anti-*c-fos *antibodies.

**Table 3:** Sequences of the Oligonucleotides used in the EMSAs.

NF-κB consensus	5'-AGTTGA**GGGGACTTTC**CCAGGC-3'	22 bp
NF-CD38	5'-AGTATG**GGGATTCCTC**AAAGAT-3'	22 bp
AP-1 consensus	5'-CGCTTGA**TGACTCA**GCCGGAA-3'	21 bp
AP1–1	5'-GGAACTC**TGAGGCA**AGGGGTT-3'	21 bp
AP1–2	5'-GCTTTTC**TGCCTCA**GAGTCTT-3'	21 bp
AP1–3	5'-CTAGCCT**AACTACA**ATTGGCC-3'	21 bp
AP1–4	5'-ATCATCT**TTGACTCAT**CTCTTTC-3'	21 bp
AP1–5	5'-CCTTCCT**TTGGTCA**GTTACAC-3'	21 bp
AP1–6	5'-CAATTCT**TGAATCA**TGCCTCT-3'	21 bp
GRE consensus	5'-TAGAGGATC**TGTACA**GGA**TGTTCT**AGAT-3'	28 bp
GRE1	5'-AATGTCACAGA**TGTTCT**CTTAATAAAGA-3'	28 bp
GRE2	5'-TTCCGAACTTC**TGTTCT**GTTTCCCTCAA-3'	28 bp
GRE3	5'-AAGCACTGCCA**TGTTCT**CACTTATAAGT-3'	28 bp
GRE4	5'-GCCATTGTAAC**TGTTCT**CCATCCTTATC-3'	28 bp

* The putative binding sites for the different transcription factors in the proximal promoter region of *cd38 *are underlined and in bold font.

### Statistical analysis

HASM cells isolated from three different donors were used in the experiments. The experiments involving EMSA and transient transfections of the constructs were repeated three times. The samples were compared by one-way ANOVA with Bonferroni's test for multiple comparisons. GraphPad PRISM statistical software program was used for statistical analyses and significance established at *P *value of ≤ 0.05.

## Results

### NF-κB, AP-1 and Glucocorticoid Receptor binding to the cd38 promoter

**Figure 1:**
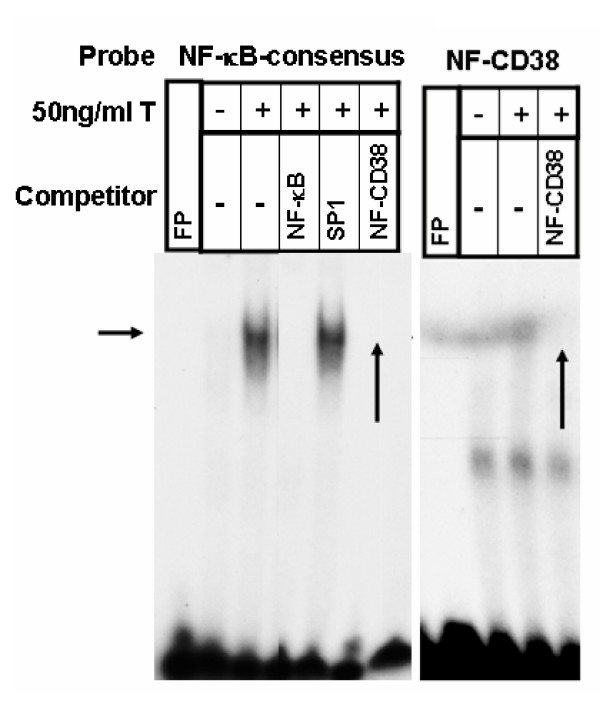
**TNF-α-induced activation and specific binding of NF-κB to the consensus and *cd*38 putative binding sites in HASM cells**. Electrophoretic mobility gel shift demonstrating binding of nuclear proteins obtained from either control (untreated) or TNF-α-treated (50 ng/ml) HASM cells to labeled oligonucleotides corresponding the consensus (NF-κB-consensus) or putative *cd38 *(NF-CD38) NF-κB binding sequences. Note NF-κB binding (indicated by horizontal arrow) in samples obtained from TNF-α-treated cells. Binding specificity was confirmed using a 100-fold excess of unlabeled oligonucleotide corresponding to either the consensus or putative sequences. Binding to the consensus and putative *cd38 *sequences is abolished by excess unlabeled putative sequence (shown by vertical arrows). SP1 oligonucleotides were used as a nonspecific competitor to confirm the specificity of the binding. FP: Free Probe in this and subsequent figures; T: TNF-α. Representative of 4 different assays.

**Figure 2:**
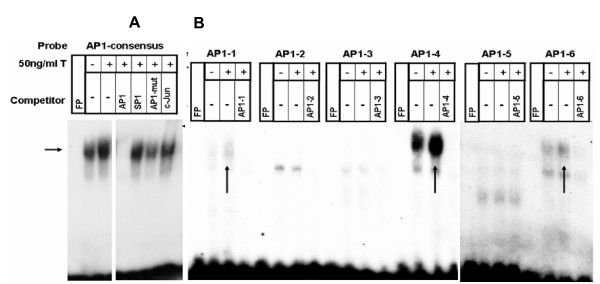
**TNF-α-induced activation of AP-1 in HASM cells**. Binding of nuclear proteins to labeled oligonucleotides corresponding to the AP-1 consensus (A) or putative *cd38 *(B) binding sequences. The specificity of binding was confirmed with excess unlabeled consensus or putative AP-1 oligonucleotide sequences as a specific competitor, and SP1 as a nonspecific competitor. Anti-*c-jun *or -*c-fos *antibodies was used for the gel supershift assay. **Panel A: **TNF-α-induced increased binding to the consensus AP-1 sequence (horizontal arrow) and gel supershift in the presence of an anti-*c-Jun *antibody (c-Jun). Note decreased binding in the presence of unlabeled consensus AP-1 (AP1) or with mutated AP-1 (AP-1 mut). **Panel B: **TNF-α-induced increased binding to labeled putative *cd38 *AP-1 sites 1, 4 and 6 (indicated by arrows and labeled AP1–1, AP1–4 and AP1–6 respectively), with the strongest binding to AP1–4.

**Figure 3:**
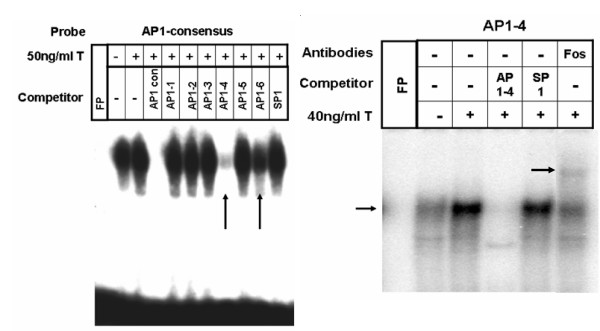
**TNF-α-induced activation and specific binding of AP-1 to the consensus and *cd*38 putative binding sites in HASM cells**. **Left Panel: **Nuclear protein binding to AP-1 consensus sequence and competition for AP-1 binding with unlabeled oligonucleotide consensus (AP-1 con) and putative AP-1 sequences (labeled AP1–1 to AP1–6). Note decreased binding with AP-1 con, and AP1–4 and AP1–6 unlabeled sequences. **Right Panel: **Nuclear protein binding to labeled oligonucleotide AP1–4 sequence (arrow on left), which is abolished in the presence of excess unlabeled oligonucleotide AP1–4 sequence (labeled AP1–4), but not by a non-specific competitor (SP1). Gel supershift with anti-*c-fos *antibodies (arrow and labeled Fos). Representative of 4 different assays.

To investigate the transcriptional regulation of CD38 expression in HASM cells, we cloned a putative 3 kb promoter fragment (GenBank Acc. No. DQ091293) from K562 cells into the pGL3 basic vector. The *cd38 *promoter sequence was examined for the presence of typical consensus elements using the GeneQuest module of Lasergene 6.0 program from DNASTAR. We identified six AP-1, one NF-κB, and four GRE motifs which are shown in Table 1. Using the electrophoretic mobility shift assay (EMSA), we examined whether transcription factors from HASM nuclear extracts or recombinant human GR proteins can bind to these putative binding sites following exposure of cells to TNF-α and dexamethasone. Oligonucleotides were synthesized from putative NF-κB, AP-1 and GRE binding sites (Table 3). The specificity of the EMSA was confirmed by competition experiments using unlabeled oligonucleotide sequences and gel supershift assays using specific antibodies. TNF-α increased the specific binding of nuclear proteins to consensus (Figure 1) as well as putative *cd38 *NF-κB sites (Figure 1), which was effectively competed with excess unlabeled consensus or putative sequences (Figure 1). EMSA also demonstrated that TNF-α increased the specific binding of nuclear proteins to the AP-1 consensus oligonucleotide sequence (Figure 2) and the putative *cd38 *AP-1 sites 1, 4 and 6 (referred to as AP1–1, AP1–4 and AP1–6 respectively), with the strongest binding to AP1–4 (Figure 2). Strong competition for binding to the consensus AP-1 sequence was observed with excess unlabeled AP1–4 sequence (Figure 3). AP-1 binding to the putative AP1–4 was confirmed by a gel supershift assay with anti-*c-fos *antibodies (Figure 3).

**Figure 4:**
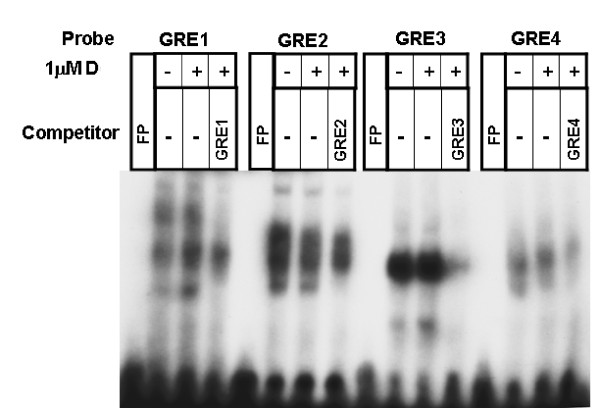
**Specific binding of GR to *cd*38 putative GRE binding sites**. Electrophoretic mobility gel shift assays demonstrating binding of nuclear proteins obtained from control or dexamethasone-treated HASM cells to labeled oligonucleotide putative *cd38 *GRE sites. To confirm specificity of binding, unlabeled oligonucleotide putative *cd38 *GRE sequences were used as a specific competitor. Dexamethasone induced binding of nuclear proteins to oligonucleotides corresponding to the *cd38 *putative GRE binding sequences 1, 3 and 4 (labeled GRE1 to GRE4), and decreased binding in the presence of the respective unlabeled oligonucleotide sequences. The binding to GRE3 is weaker compared to the other putative GRE motifs. Note that there is no increase in nuclear protein binding to GRE2 by dexamethasone compared to untreated control.

**Figure 5:**
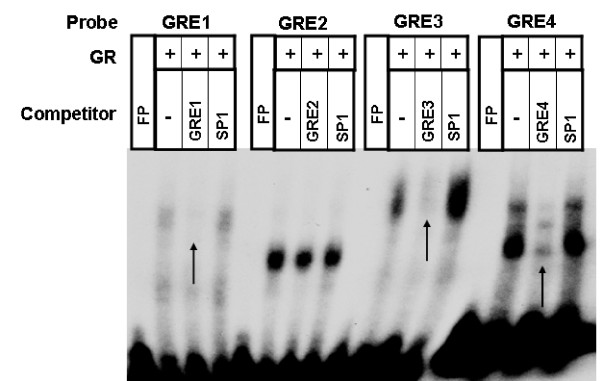
**Binding of recombinant glucocorticoid receptor (GR) to *cd*38 putative GRE sequences**. Binding of recombinant glucocorticoid receptor (GR) to *cd38 *putative GRE sequences showing increased binding to GRE sequences 1, 3 and 4, and competition for binding with the respective unlabeled oligonucleotide sequences (indicated by arrows).

**Figure 6:**
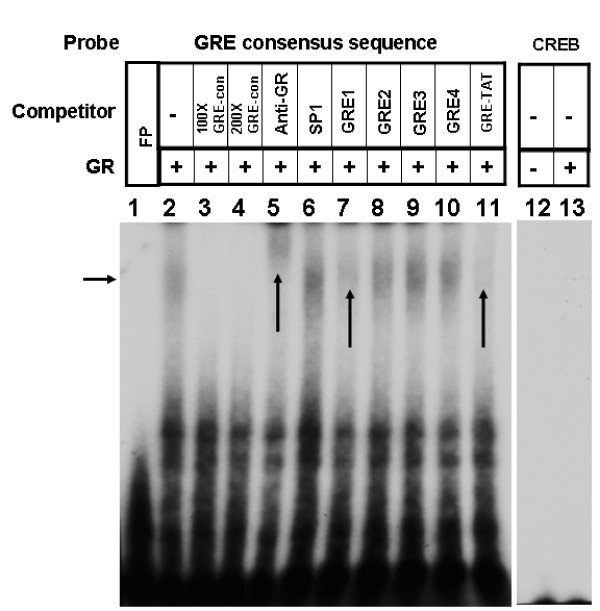
**Binding of recombinant glucocorticoid receptor (GR) to consensus GRE sequences**. Binding of recombinant GR to labeled consensus GRE sequence (lane 2 and indicated by horizontal arrow), competition for binding with *cd38 *putative GRE sequences (labeled GRE1 to GRE4, lanes 7–10), and gel supershift with anti-GR antibodies (Anti-GR, lane 5). Note decreased binding in the presence of either 100- (100 × GRE-con, lane 3) or 200- (200 × GRE-con, lane 4) fold excess unlabeled consensus sequence or 100-fold GRE-TAT (lane 11, vertical arrow), a known GRE binding sequence. Competition assays with excess unlabeled *cd38 *putative GRE sequences reveal decreased binding only in the presence of the GRE1 (lane 7, vertical arrow). Note gel supershift in the presence of an anti-GR antibody (shown as anti-GR). Lanes on extreme right show no specific binding of GR to an irrelevant binding site (shown here for CREB, lanes 12 and 13). Representative of 4 assays.

Glucocorticoid receptor (GR) binding to consensus GRE and putative GREs from *cd38 *sequences were performed using nuclear extract obtained from dexamethasone-treated HASM cells. Dexamethasone increased the binding of nuclear proteins to putative *cd38 *GRE sites 1, 3 (slight increase) and 4, but not to the GRE site 2 (Figure 4). This binding was inhibited with the respective excess unlabeled oligonucleotide sequences. To examine the direct binding of GR to putative GRE sites, we performed EMSA with recombinant human GR protein. There was binding of recombinant GR to labeled oligonucleotide putative *cd38 *GRE sites 1, 3 and 4 (Figure 5) as well as consensus GRE sequence (Figure 6). The binding of GR to the putative *cd38 *GRE sites 1, 3 and 4 was inhibited by excess unlabeled oligonucleotide sequences (Figure 5). Furthermore, the GR binding to the labeled consensus GRE sequence was inhibited by excess unlabeled *cd38 *putative GRE1, but not by the other putative GRE sequences (Figure 6) as well as by GRE-TAT, a GRE site from tyrosine aminotransferase gene (Figures 6). There was no binding of GR to an irrelevant sequence, as shown by a lack of binding to CREB binding sites (Figure 6). The specificity of GR binding to the consensus GRE sequence was further substantiated by gel supershift with an anti-GR antibody. The EMSA with HASM nuclear extract and putative GRE sites showed several binding complexes (Figure 4), which is not unexpected since GR is known to interact with many co-activators in the nucleus [15,16].

### Activation of the cd38 promoter requires NF-κB and AP-1, and is inhibited by dexamethasone

**Figure 7:**
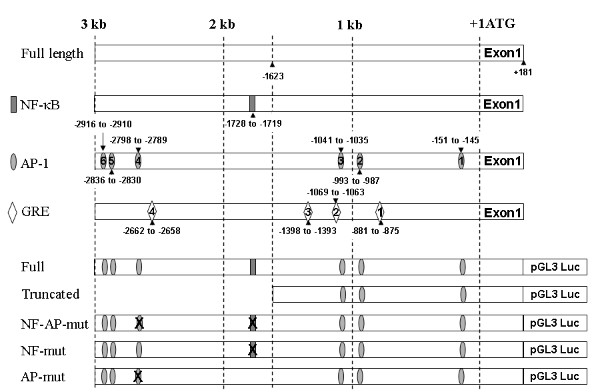
**The cloned 3 kb *cd38 *promoter showing the location of the putative binding sites for NF-κB, AP-1 and GR (labeled NF-κB, AP-1 and GRE)**. Location of the putative binding sites for NF-κB, AP-1, and GRE on the *cd38 *promoter, the 3 kb (Full) promoter, the truncated 1.8 kb promoter (Truncated), and promoter constructs with mutations in the binding sites for NF-κB or AP1–4 or both binding sites (NF-AP-mut, NF-mut and AP-mut). The promoter was cloned in front of a luciferase reporter gene in the pGL3 plasmid and was used to transfect HASM cells.

**Figure 8:**
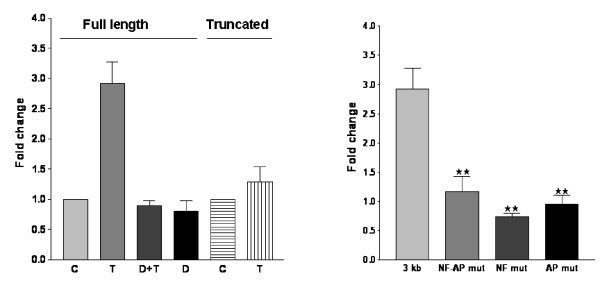
**Activation of the *cd38 *promoter in the HASM cells**. Luciferase activity was measured as an index of promoter activation with the Renilla luciferase activity (pRL-TK) to normalize for transfection efficiency. The normalized luciferase activity is expressed as the fold change compared to the control. **Left Panel: **Activation of the full length promoter and the truncated promoter. TNF-α (T) causes activation of the 3 kb promoter as compared to control (C), which is inhibited in the presence of dexamethasone (D+T). Truncated promoter: There is no activation of the truncated promoter by TNF-α. **Right Panel: **TNF-α causes activation of the 3 kb promoter (3 kb), but not the constructs with mutations in NF-κB or/and AP-1 site 4 (labelled NF-AP mut, NF mut and AP mut). Representative of 3 different assays.

The EMSA studies revealed that TNF-α increased the binding of nuclear proteins to the putative NF-κB site, and to some of the putative AP-1 sites in the *cd38 *promoter. Likewise, dexamethasone increased the binding of nuclear proteins selectively to some of the putative *cd38 *GREs. To investigate whether TNF-α modulates *cd38 *promoter activity directly, HASM cells were transiently transfected with a *cd38 *promoter-driven luciferase reporter construct. In the initial studies, we used the 3 kb promoter (Figure 7) and a truncated 1.8 kb promoter that lacks the NF-κB site and the AP1–4 site that exhibited very strong binding following TNF-α treatment. HASM cells were transiently transfected with the promoter constructs and luciferase activity was determined following exposure to TNF-α. TNF-α caused an increase in luciferase activity of the 3 kb promoter, but not the truncated 1.8 kb promoter, and dexamethasone decreased the TNF-α-induced activation of the 3 kb promoter (Figure 8)). To determine the transcription factor binding sites within the 3 kb promoter that are involved in the regulation of CD38 expression, HASM cells were transfected with site directed mutated constructs. For these studies, *cd38 *promoter luciferase constructs mutated at the NF-κB site or the AP1–4 site, or at both of these sites were used. Following exposure to TNF-α, luciferase activity was abolished in the promoter constructs with mutations of either the NF-κB or the AP1–4 sites, or mutation in both the sites (Figure 8). The EMSA results and the decreased activation of the promoter with mutations (that lack the NF-κB and the dominant AP1–4 binding sites) confirm a functional role for NF-κB and AP1–4 in the transcriptional regulation of CD38. Glucocorticoid regulation also involves binding to *cd38 *GREs and inhibition of NF-κB- and AP-1-dependent transcription.

## Discussion

Airway hyperresponsiveness to non-specific stimuli is a hallmark of asthma. In this regard, airway smooth muscle has a role in the regulation of airflow and in maintaining airway caliber. Airway smooth muscle contractility requires the elevation of intracellular calcium and the CD38/cADPR signaling pathway has a central role in calcium homeostasis [7]. A previous study from our laboratory demonstrated that CD38 expression is up-regulated by the proinflammatory cytokine TNF-α resulting in an increased intracellular calcium response to multiple agonists [5]. The increased CD38 expression is down-regulated by the anti-inflammatory glucocorticoid dexamethasone through inhibition of NF-κB [9]. In this study, we characterized a 3 kb fragment that functions as a promoter of the *cd38 *gene. We also show that the *cd38 *promoter contains one NF-κB, six AP-1, and four GRE putative binding sites. TNF-α caused activation of the 3 kb promoter fragment, which is decreased when the NF-κB and/or the AP1–4 sites were mutated. The EMSA studies confirmed direct binding of NF-κB and AP-1 to putative *cd38 *binding sites. Dexamethasone reversed the TNF-α-induced activation of the 3 kb promoter and increased the binding of GR to consensus and putative *cd38 *GREs. These studies demonstrate an important role of NF-κB and AP-1 in the regulation of CD38 expression in HASM cells. Furthermore, glucocorticoids decrease CD38 expression transcriptionally by directly binding to the putative *cis*-acting binding sites and also by interfering with the transcription factors.

The *cd38 *gene has been localized on chromosome 4 in human and chromosome 5 in the mouse [17]. The CD38 protein is encoded by a >80 kb length gene comprising of 8 exons. Studies from other laboratories have revealed binding sites for several transcription activating factors in the *cd38 *gene [17,18]. Previous studies have shown the absence of a canonical TATA or CAAT box sequences in the *cd38 *promoter region, suggesting that transcription can be initiated at multiple sites [19]. However, TATA-less promoters with transcription start sites such as an initiator (Inr) sequence or binding sites for the PU.1 transcription factor have been described in myeloid and B cells [20]. The G/C rich region upstream of ATG may also support the initiation of transcription. In addition, consensus binding sites for T cell transcription factor (TCF-1α), Ig gene box enhancer motifs (μE1, μE5 and κE2), nuclear factor-IL-6 and IFN-responsive factor-1 have been described [21]. Kishimoto *et al *[13] have reported the DR5 repeat (TGACCCgaaagTGCCCC) within intron 1, which has a role in retinoic acid induction of CD38 expression in HL-60 cells. Studies from other laboratories have revealed a ~900 bp CpG island spanning exon 1 and the 5' end of intron 1 with a binding sequence for Sp1, a transcription factor that regulates the constitutive expression of CD38 [22]. Furthermore, a glucocorticoid response element and an estrogen binding motif have also been described in the promoter region of *cd38 *[22]. In support of a functional role of the estrogen binding motif within the promoter, our previous studies demonstrate the up-regulation of CD38 expression by estrogen in uterine smooth muscle [23-25]. Taken together, it is likely the transcriptional regulation of CD38 expression by these hormones may have a physiological role in uterine motility.

Inflammatory cytokines such as TNF-α, IL-1β and IFN-γ play an important role in diseases such as asthma [26,27]. Previous investigations have demonstrated that the levels of inflammatory cytokines are elevated in the bronchoalveolar lavage fluid obtained from asthmatic subjects [26,27]. TNF-α has been shown to increase the expression of a variety of genes resulting in functional changes in airway smooth muscle cells [28,29]. Recent investigations from our laboratory have shown that the inflammatory cytokines increase the expression of CD38 in human airway smooth muscle cells [5,7,8]. The regulation of CD38 expression by TNF-α in HASM cells involves NF-κB and AP-1 activation and signaling through the p38 and JNK MAP kinases [9,10]. TNF-α-induced CD38 expression in airway smooth muscle cells involves signaling via the TNFR1 receptor and IFNβ that is generated in response to TNF-α [30]. Thus, the induction of CD38 expression by TNF-α may involve regulation by multiple transcription factors such as interferon regulatory factor-1, NF-κB, AP-1 and possibly others. In this context, sequence analysis of the cloned human *cd38 *promoter also reveals 4 putative binding sites for the transcription factor c/EBPβ, three of which are within a region upstream of the NF-κB site. The 1.8 kb truncated promoter construct that was not activated by TNF-α also contains these c/EBPβ sites. The role, if any, of this transcription factor in the regulation of CD38 expression in HASM cells remains to be determined.

Glucocorticoids are used extensively as anti-inflammatory therapy in asthma [11] and their mechanism(s) of action are complex [31]. The nuclear translocation of the GR complex and its binding to specific DNA motifs results in both transactivation and repression of a variety of genes [12,32-34]. The presence of GREs provides a basis for transcriptional regulation of CD38 expression. The GR complex also interferes with NF-κB binding to DNA [35,36]., thereby decreasing the expression of genes that are regulated by this transcription factor. We have previously demonstrated inhibition of NF-κB activation by dexamethasone in HASM cells exposed to TNF-α [9]. This inhibition results from decreased NF-κB expression and increased IκB expression following exposure to dexamethasone. This mechanism of regulation of NF-κB activation has been described in other cell systems [33,37]. In preliminary studies, we have also noticed decreased AP-1 activation in TNF-α-stimulated cells by dexamethasone. The mechanism of glucocorticoid-mediated reduction of CD38 expression may involve steric hindrance for the binding of NF-κB and AP-1 to their binding sites and/or interference with transactivation. The actions of glucocorticoids have been demonstrated for the NF-κB- and AP-1-mediated regulation of other genes [34,38-43].

In this study, we have identified 4 glucocorticoid response elements in the putative promoter region of the *cd38 *gene as well as response elements for AP-1 and NF-κB (Table 1). Inhibition of NF-κB or AP-1 activation, or MAPK signaling using pharmacological and molecular tools has confirmed their role in the regulation of CD38 expression [9,10]. The identified putative sites for AP-1 and GRE also exhibit strong binding in EMSA upon exposure to TNF-α and dexamethasone respectively. The AP1–4 site (residing between -2798 to -2789 bp) that shows very strong binding also appears to be functionally important in the activation of the promoter, since mutation of this site profoundly affected TNF-α-induced activation of CD38 expression. With respect to NF-κB, mutation of the only identifiable binding site also resulted in abolition of CD38 transcription. It is worth noting that binding to this site was weak compared to the consensus NF-κB sequence binding, although competition with the unlabelled putative sequence effectively abolished the strong binding to the consensus sequence. In the presence of dexamethasone, there was complete reversal of TNF-α-induced activation of the promoter, indicating direct transcriptional regulation of CD38 expression by glucocorticoids in HASM cells. These findings implicate the importance of NF-κB and AP-1, and the GRE within the proximal promoter region in the regulation of CD38 gene expression. The results of promoter transfections and EMSAs with *cd38 *putative GREs demonstrate transcriptional repression of CD38 expression by glucocorticoids. However, glucocorticoids are also known to repress gene expression in HASM cells through inhibition of histone acetylation [44]. Evidence for glucocorticoid resistance of CD38 expression in HASM cells has also been reported when a combination of cytokines is used as the stimulus as opposed to the single stimulus used in the present study. In this context, a recent study showed that in the combined presence of TNF-α and IFN-γ or IFN-β, CD38 expression in HASM cells becomes refractory to glucocorticoids [45]. The mechanism appears to involve induction of the dominant negative GR-β. Thus, the glucocorticoid regulation of CD38 expression in airway smooth muscle cells is very complex and appears to depend on the stimulus or combination of stimuli used.

In a recent study, Sun *et al *described the structure of the promoter region of rabbit *cd38 *and provided evidence for the functional regulation of CD38 expression in osteoblast and osteoclast cell lines [14]. In a region encompassing 1.5 kb of the promoter obtained from a rabbit genomic DNA library, the authors identified potential binding sites for SP-1, AP-1, and AP-4. Using promoter-reporter assays similar to those described in the present studies, with a 1.5 kb promoter and several deletion mutants, they were able to demonstrate a functional AP-1 site in the 1.0 kb promoter fragment. There also appears to be cell-type specific activation of the promoter as shown by studies with deletion mutagenesis.

## Conclusion

In the present study, we describe NF-κB and AP-1 binding motifs within the *cd38 *promoter that exhibit very strong binding of nuclear proteins, mutations of which decrease promoter activation and hence may be functionally relevant. Our results also support the role of multiple transcription factors in the regulation of CD38 expression in HASM cells. Furthermore, we demonstrate a direct transcriptional control of CD38 expression by glucocorticoids, although we have not identified specific GREs within the proximal promoter region involved in this regulation. The fact that CD38 expression is regulated by cytokines and transcription factors that are implicated in asthma, and inhibited by glucocorticoids which are a mainstay of asthma therapy makes this an attractive therapeutic target.

## Competing interests

The author(s) declare that they have no competing interests.

## Authors' contributions

KGT and BNK contributed equally to the studies and should be considered co-first authors. KGT cloned the human *cd38 *promoter fragments and carried out the sequence alignment. BNK carried out the electrophoretic mobility shift assays and the promoter activation assays. Both KGT and BNK drafted the manuscript. DNF, TFW and MSK conceived of the investigations, helped in the design of the experiments, and helped to draft the final manuscript. RAP participated in the study by providing well-characterized human airway smooth muscle cells and helped in the draft of the manuscript.
